# Decency toward Horses

**Published:** 1881-06

**Authors:** 


### DECENCY TOWARD HORSES.

An old and extensive horseman says: A horse cannot be screamed at and cursed without becoming less valuable in every particular. To reach the highest degree of value, the animal should be perfectly gentle and always reliable, but if it expects every moment that it is in harness to be "jawed" at and struck, it will be in a constant state of nervousness, and in its excitement liable to do something through fear which is unexpected as to go along doing what you started to do. It is possible to train a horse to be governed by a word, almost as completely as it is to train a child, and in such training the horse reaches its highest value. When a horse is soothed by the gentle words of his driver—and we have seen him calmed down from great excitement by no other means—it may be very fairly concluded that he is a valuable animal for all practical purposes, and it may be certainly concluded that the man who has such power over him is a humane man and a sensible one. But all this simply means that the man must secure the animal's confidence. Only in exceptional instances is a horse stubborn or vicious. If he understands his surroundings, and what is required of him, he gives no trouble. As almost every reader must know, if the animal, when frightened, can be brought up to the object he will become calm. The reason is that he understands there is nothing to fear. So he must be taught to have confidence in the man who handles him, and then this powerful animal, which usually no man could handle, if he were disposed to be vicious, will give no trouble. The best rule, therefore, which we would lay down for the management of the horse, is gentleness and good sense on the part of the driver. Bad drivers make bad horses usually.

### PECULIAR INDUSTRIES.

Among the many peculiar industries is making an imitation honey in the comb. The comb is molded out of paraffine wax, in imitation of the work of bees; the cells are then filled with simple glucose syrup, flavored with some genuine honey, and sealed up by passing a hot iron over them. The product is sold for the best clover honey, and much of it is shipped to Europe. Confectioners employ glucose very largely as a substitute for cane sugar, and likewise use immense quantities of white earth (*terra alba*), which, being very cheap, is used to make weight and bulk.

Tomato catsup is made from the skins and refuse of the great tomato-canning establishments.

